# Spiropyran-Isoquinoline Dyad as a Dual Chemosensor for Co(II) and In(III) Detection

**DOI:** 10.3390/molecules22091569

**Published:** 2017-09-19

**Authors:** Yong-Min Kho, Eun Ju Shin

**Affiliations:** Department of Chemistry, Sunchon National University, Suncheon, Jeonnam 57922, Korea; melon414@scnu.ac.kr

**Keywords:** spiropyran, isoquinoline, chemosensor, cobalt, indium

## Abstract

Spiropyran derivatives have been studied as light-regulated chemosensors for a variety of metal cations and anions, but there is little research on chemosensors that simultaneously detect multiple metal cations. In this study, a spiropyran derivative with isoquinoline, **SP-IQ**, was prepared and it functions investigated as a light-regulated sensor for both Co^2+^ and In^3+^ cations. A colorless nonfluorescent **SP-IQ** converts to a pink-colored fluorescent **MC-IQ** by UV irradiation or standing in the dark, and **MC-IQ** returns to **SP-IQ** with visible light. Upon UV irradiation with the Co^2+^ cation for 7 min, the stronger absorption at 540 nm and the similar fluorescence intensity at 640 nm are observed, compared to when no metal cation is added, due to the formation of a Co^2+^ complex with pink color and pink fluorescence. When placed in the dark with the In^3+^ cation for 7 h, the colorless solution of **SP-IQ** changes to the In^3+^ complex with yellow color and pink fluorescence, which shows strong absorption at 410 nm and strong fluorescence at 640 nm. Selective detection of the Co^2+^ cation with UV irradiation and the In^3+^ cation in the dark could be possible with **SP-IQ** by both absorption and fluorescence spectroscopy or by the naked eye.

## 1. Introduction

Cobalt salts have been used as pigments since ancient times to produce brilliant blue colors in paint, porcelain, and glass [1,2]. Cobalt alloys are used in powerful magnets, jet turbines, and medical orthopedic implants of the hip and knee because of its high-temperature strength, and corrosion and wear resistance. Radioactive cobalt-60 is used to treat cancer in radiotherapy. Cobalt is an essential trace element as a component of vitamin B_12_. However, cobalt is carcinogenic and causes heart and lung problems, occupational asthma, and vision and hearing problems, including tinnitus, deafness, and blindness [3].

Indium is notably used in the semiconductor industry to make indium tin oxide (ITO) for transparent conductive coatings on glass, which is an important part of touch screens, flat-screen TVs, and solar panels [4,5]. Indium metal is used as a light filter in low-pressure sodium vapor lamps, and as a mirror finish on windows of tall of an embryo to be teratogenic [6].

Numerous chemosensors have been buildings. Indium compounds are highly toxic and damage the heart, kidney, and liver, and causes the development developed for sensing various metal cations and anions. Among those, the detection techniques based on the change of color and/or fluorescence provide simple and inexpensive tools [7,8]. Therefore, colorimetric and/or fluorescent chemosensors for the detection of toxic heavy metals, such as Co^2+^ and In^3+^, have attracted significant interest because of their potential application in chemical, biological, industrial, and environmental research.

Organic photochromic materials have received great attention in various applications, including light-tunable biological and chemical systems, molecular logic gates, and light-regulated chemosensors [9]. Photochromic materials change their color reversibly through structural changes depending on whether they are exposed to UV light.

**Scheme 1:**
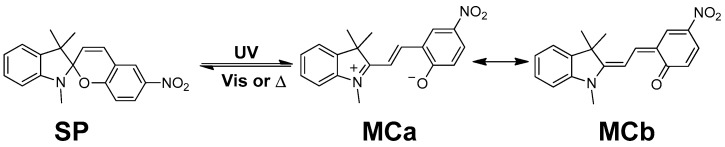
Interconversion between spiropyran (**SP**) and merocyanine (**MC**). **MC** is a resonance hybrid between **MCa** and **MCb**.

Spiropyran derivatives have been extensively studied as typical photochromic molecules, which accomplish the reversible interconversion between colorless non-fluorescent spiropyran and colored fluorescent merocyanine (Scheme 1) [10]. Upon UV irradiation, a ring-closed spiropyran form is converted into a ring-opened merocyanine form by heterolytic cleavage of the spiro C–O bond [11]. Merocyanine is a resonance hybrid between a zwitterion form **MCa** and a neutral quinonoid form **MCb** [12,13]. The merocyanine form is returned to the spiropyran form by ring-closure when exposed to visible light or thermal energy.

A negatively-charged phenolic oxygen in the ring-opened merocyanine form provides a chelating site for a variety of metal cations [14]. Spiropyran derivatives appended with a suitable ligand, which provides a cooperative binding site along with negatively-charged phenolic oxygen, can lead to light-regulated sensors for metal cations [15]. A variety of spiropyran-based chemosensors has been investigated as a selective, sensitive, and reproducible detection system for various metal cations [13,14,15,16,17,18].

Although chemosensors for a specific metal cation have been extensively developed, there are few studies on chemosensors that simultaneously detect multiple analytes [19,20,21].

In this paper, we report that **SP-IQ**, a spiropyran derivative appended with isoquinoline, functions as a light-regulated sensor for both Co^2+^ and In^3+^ cations.

## 2. Results and Discussion

### 2.1. Interconversion between **SP-IQ** and **MC-IQ**

**SP-IQ** is colorless and non-fluorescent. It shows absorption maxima at 272 and 325 nm in CH_3_CN/H_2_O (1/1, *v*/*v*).

**Figure 1:**
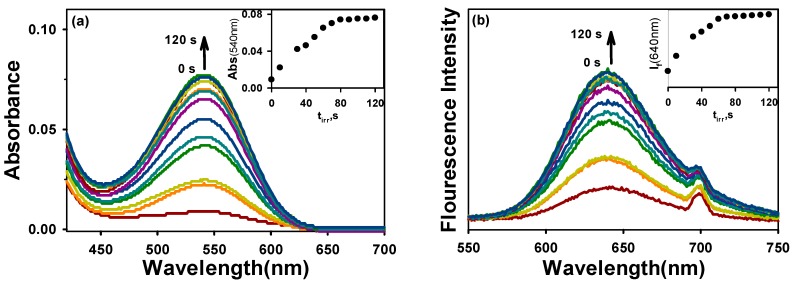
(**a**) Absorption and (**b**) fluorescence spectral changes of **SP-IQ** with irradiation time (0–120 s, Different color lines represent different irradiation times.) with UV irradiation at 350 nm. Inset: Plots of (**a**) absorbance at 540 nm and (**b**) fluorescence intensity at 640 nm vs. irradiation time.

Irradiation of **SP-IQ** using 350 nm light changed the colorless solution to **MC-IQ** of pink color. Absorption and fluorescence spectral changes of **SP-IQ** in CH_3_CN/H_2_O (1/1, *v*/*v*) with the irradiation at 350 nm are shown in Figure 1. Due to the photoinduced conversion by C–O bond cleavage from **SP-IQ**, a spiropyran form, to **MC-IQ**, a merocyanine form, new absorption at 540 nm and new fluorescence at 640 nm increase with irradiation time.

**Figure 2:**
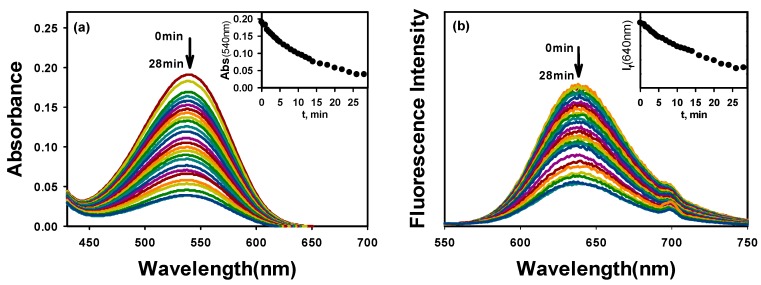
(**a**) Absorption and (**b**) fluorescence spectral changes by reverse reaction from **MC-IQ** (formed after irradiation of **SP-IQ** with 350 nm light for 7 min) to **SP-IQ** over time (0–28 min, Different color lines represent different irradiation times.) under room illumination. Inset: Plots of (**a**) absorbance at 540 nm and (**b**) fluorescence intensity at 640 nm vs. room illumination time.

Figure 2 shows the spectral changes of absorption and fluorescence by the reverse reaction from **MC-IQ** (formed after irradiation of **SP-IQ** with 350 nm light for 7 min) to **SP-IQ** over time (0–28 min) under room illumination. When placed under room light, the pink color of **MC-IQ** is blurred. As **MC-IQ** reverts to **SP-IQ** through a ring closure reaction by visible light, the absorbance at 540 nm and the fluorescence intensity at 640 nm decrease.

**Figure 3:**
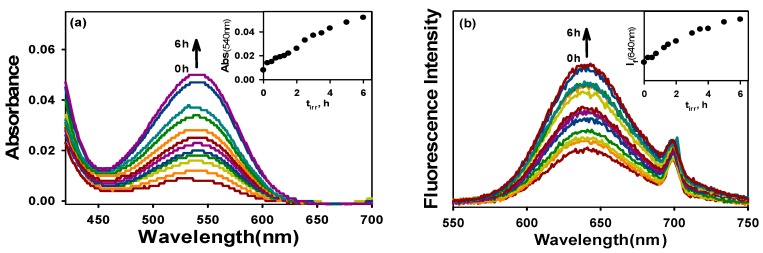
(**a**) Absorption and (**b**) fluorescence spectral changes of **SP-IQ** over time (0–6 h, Different color lines represent different times.) during the dark incubation. Inset: Plots of (**a**) absorbance at 540 nm and (**b**) fluorescence intensity at 640 nm vs. irradiation time.

The absorption and fluorescence spectral changes of **SP-IQ** which is placed in the dark (Figure 3) are very similar to those of **SP-IQ** which is irradiated at 350 nm. The new absorption at 540 nm and the new fluorescence at 640 nm slowly increase over 6 h when the solution of **SP-IQ** is placed in the dark. The ring-opening reaction from **SP-IQ** to **MC-IQ** is thought to occur not only on UV irradiation, but also in the dark. Probably, the major isomer in UV light or darkness is **MC-IQ**, whereas the major isomer under visible light is **SP-IQ**.

**Figure 4:**
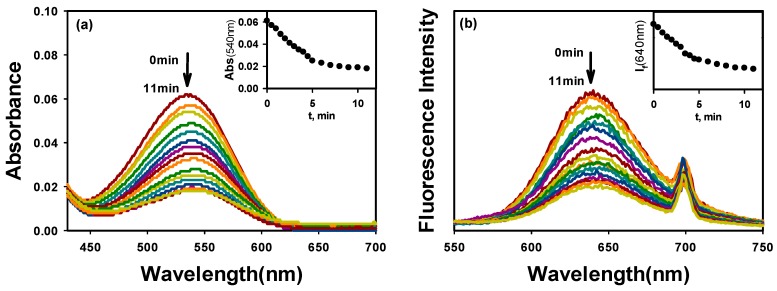
(**a**) Absorption and (**b**) fluorescence spectral changes by reverse reaction from **MC-IQ** (formed after dark incubation of **SP-IQ** for 7 h) to **SP-IQ** with time (0–11 min, Different color lines represent different times.) under room illumination. Inset: Plots of (**a**) absorbance at 540 nm and (**b**) fluorescence intensity at 640 nm vs. room illumination time.

Figure 4 shows the spectral changes of absorption and fluorescence by reverse reaction from **MC-IQ** (formed after dark incubation of **SP-IQ** for 7 h) to **SP-IQ** with time (0–11 min) under room illumination. When placed in a lit room, the absorbance at 540 nm and the fluorescence intensity at 640 nm decrease, indicating that **MC-IQ** returns to **SP-IQ** through the ring closure reaction by visible light.

**Scheme 2:**
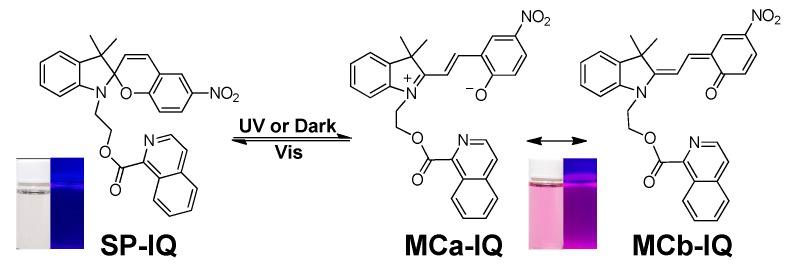
Interconversion between **SP-IQ** and **MC-IQ**. **MC-IQ** is a resonance hybrid between **MCa-IQ** and **MCb-IQ**. In each pair of photos, left and right show the color and fluorescence of the solution, respectively.

Most spiropyran derivatives convert from the spiropyran form to the merocyanine form by UV irradiation. Conversely, merocyanine converts to spiropyran by visible light, or thermally [10]. However, a colorless nonfluorescent **SP-IQ** converts to a pink-colored fluorescent **MC-IQ** by UV irradiation or in the dark, and **MC-IQ** returns to **SP-IQ** by visible light (Scheme 2). Regardless of whether **MC-IQ** is formed by UV irradiation or in the dark, **MC-IQ** returns to **SP-IQ** under room illumination, i.e., visible light. These observations are unusual and interesting. It is thought that while **SP-IQ** is the preferred isomer under visible light, **MC-IQ** is the preferred isomer in UV light or in the dark.

### 2.2. Selective Sensing of Co^2+^

Absorption and fluorescence spectra of **SP-IQ** show no changes with adding various metal cations (Ag^2+^, Al^3+^, Cd^2+^, Co^2+^, Cu^2+^, Cu^+^, Fe^2+^, Hg^2+^, In^3+^, Ni^2+^, Zn^2+^).

**Figure 5:**
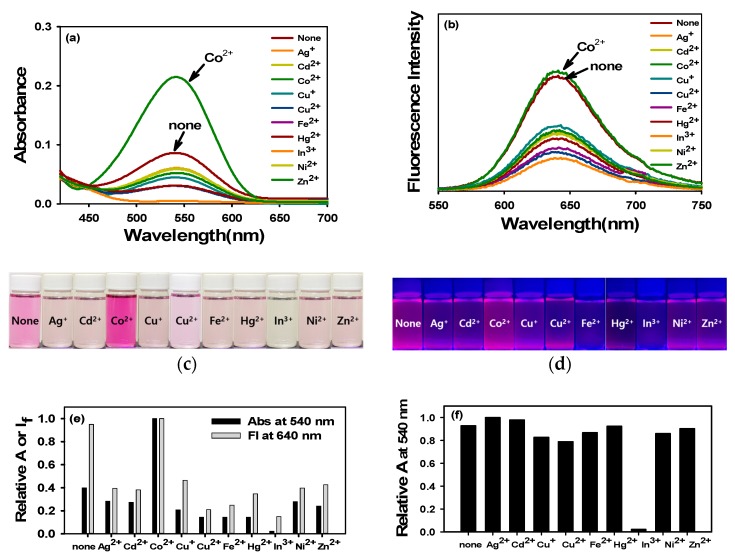
(**a**) Absorption spectra, (**b**) fluorescence spectra, (**c**) photograph of visual color change, (**d**) photograph of fluorescence change, and (**e**) relative absorbance and fluorescence intensity of **SP-IQ** after 350 nm UV irradiation for 7 min with the addition of various metal cations; and (**f**) relative absorbance of **SP-IQ** after 350 nm UV irradiation for 7 min with the addition of various metal cations (1 × 10^−4^ M; Ag^2+^, Cd^2+^, Cu^+^, Cu^2+^, Fe^2+^, Hg^2+^, In^3+^, Ni^2+^, Zn^2+^) in the presence of Co^2+^ (1 × 10^−4^ M).

However, upon UV irradiation, some changes for the absorption and fluorescence spectra of **SP-IQ** are observed with the addition of various metal cations. Figure 5 shows the absorption and fluorescence spectra, and the visual color and fluorescence of **SP-IQ** after 350 nm UV irradiation for 7 min with the addition of various metal cations. Figure 5a,b show that an absorption band at 540 nm and a fluorescence band at 640 nm appear after UV irradiation of **SP-IQ** without metal cations. UV irradiation with the Co^2+^ cation exhibits a high absorbance at 540 and a similar fluorescence intensity at 640 nm, compared to the absence of metal cations. In the presence of metal cations other than Co^2+^ cation, the absorption at 540 nm and the fluorescence at 640 nm become weaker. Figure 5c shows pink solutions with no metal cation and with the addition of Co^2+^ cation. Colorless and nonfluorescent **SP-IQ** converts to pink **MC-IQ** after UV irradiation without metal cations. After UV irradiation with the addition of the Co^2+^ cation, the solution shows a pink color and pink fluorescence due to the formation of a complex **MCa-IQ–Co^2+^** between **MCa-IQ** and the Co^2+^ cation. In the presence of metal cations other than Co^2+^ cation, the pink color becomes pale. The solution is colorless in the presence of the In^3+^ cation. Figure 5d shows pink fluorescence with no metal cation and with the addition of Co^2+^ cation. In the presence of other metal cations other than Co^2+^ cation, the fluorescence is weaker. The fluorescence is strongly quenched in the presence of In^3+^ cation. As shown in Figure 5e, stronger absorption at 540 nm and similar fluorescence intensity at 640 nm are observed with the Co^2+^ cation, compared to when no metal cation is added. In the presence of metal cations other than the Co^2+^ cation, the absorption at 540 nm and the fluorescence at 640 nm is weaker. Figure 5f shows that the existence of other competing ions, except the In^3+^ cation, does not disturb the selective detection of the Co^2+^ cation.

**Figure 6:**
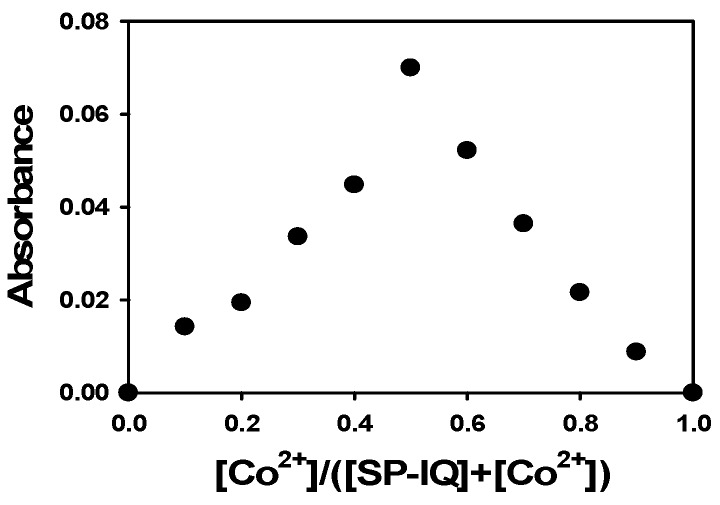
Job plot of **SP-IQ** and Co^2+^ cation. Absorbance at 540 nm for the mixed solutions of **SP-IQ** and Co^2+^ cation after 350 nm UV irradiation for 7 min was plotted as a function of the molar ratio [Co^2+^]/([**SP-IQ**] + [Co^2+^]). The total concentration for the mixed solutions of **SP-IQ** and the Co^2+^ cation was 1 × 10^−5^ M.

The Job plot indicates the formation of a 1:1 complex between **SP-IQ** and the Co^2+^ cation (Figure 6).

**Figure 7:**
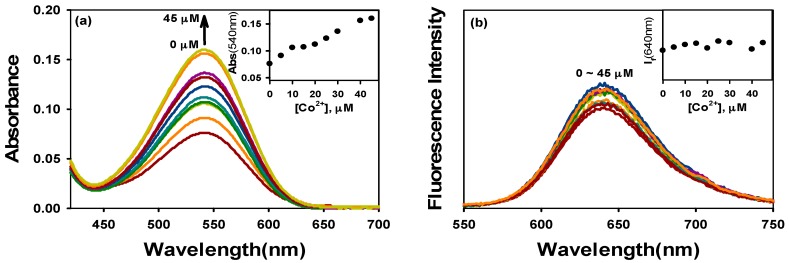
(**a**) Absorption and (**b**) fluorescence spectra of **SP-IQ** after 350 nm UV irradiation for 7 min with the various concentration of Co^2+^ (0~45 μM, Different color lines represent different Co^2+^ concentrations.). Inset: Plots of (**a**) absorbance at 540 nm and (**b**) fluorescence intensity at 640 nm vs. [Co^2+^].

Figure 7 shows the absorption and fluorescence spectra of **SP-IQ** after 350 nm UV irradiation for 7 min with the various concentrations of Co^2+^ cations. **MC-IQ** formed by 350 nm UV irradiation for 7 min produces **MCa-IQ–Co^2+^** complex with the Co^2+^ cation. As the concentration of Co^2+^ increases, more **MCa-IQ–Co^2+^** complex are formed and the absorbance at 540 nm linearly increases up to two-fold. However, the fluorescence intensity at 640 nm does not change significantly even when the concentration of Co^2+^ increases. It is thought that the fluorescence efficiency of **MCa-IQ–Co^2+^** complexes are similar with that of **MC-IQ**.

### 2.3. Effect of Other Metal Cations Except Co^2+^—in the Case of Fe^2+^

**Figure 8:**
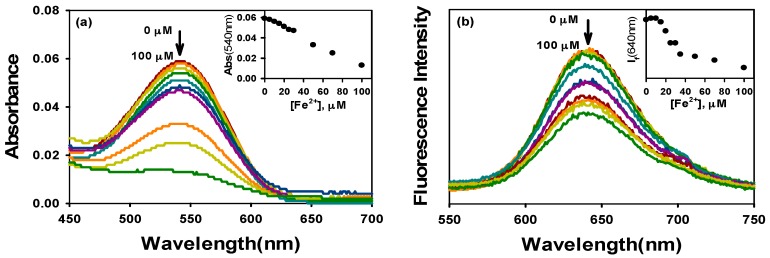
(**a**) Absorption and (**b**) fluorescence spectra of **SP-IQ** after 350 nm UV irradiation for 7 min with various concentration of Fe^2+^ (0~100 μM, Different color lines represent different Fe^2+^ concentrations.). Inset: Plots of (**a**) absorbance at 540 nm and (**b**) fluorescence intensity at 640 nm vs. [Fe^2+^].

To investigate the changes in absorption and fluorescence in the presence of other metal cations except Co^2+^, we chose the Fe^2+^ cation arbitrarily among the cations. The absorption and fluorescence spectra of **SP-IQ** after 350 nm UV irradiation for 7 min with various concentration of Fe^2+^ are shown in Figure 8. Both absorption at 540 nm and fluorescence at 640 nm are weakened with the increased concentration of Fe^2+^, in contrast that these are enhanced with the increased concentration of Co^2+^. The addition of Fe^2+^ and other metal cations except Co^2+^ is presumed to inhibit the photoconversion from **SP-IQ** to **MC-IQ** by UV irradiation.

This result shows that the Co^2+^ cation can be selectively detected among the cations.

### 2.4. Selective Sensing of In^3+^

**Figure 9:**
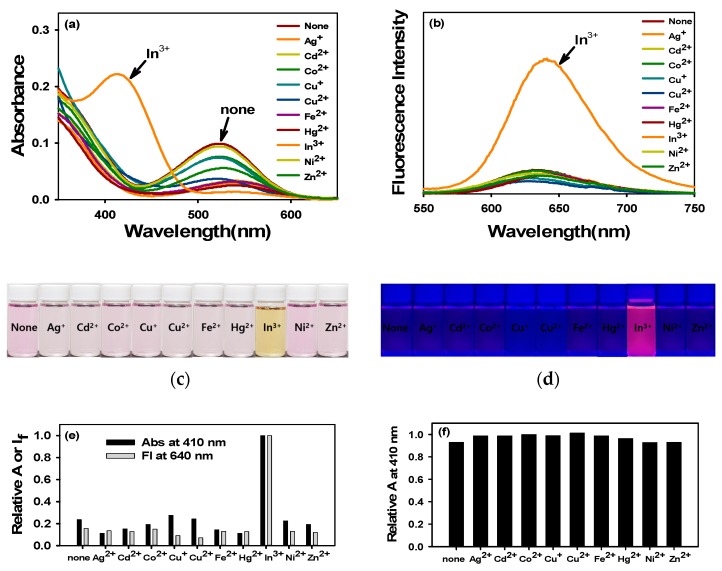
(**a**) Absorption spectra, (**b**) fluorescence spectra, (**c**) photograph of visual color change, (**d**) photograph of fluorescence change, and (**e**) relative absorbance and fluorescence intensity of **SP-IQ** after placed in the dark for 7 h with the absence and the presence of various metal cations; and (**f**) relative absorbance of **SP-IQ** after being placed in the dark for 7 h with the absence and the presence of various metal cations (1 × 10^−4^ M; Ag^2+^, Cd^2+^, Co^2+^, Cu^+^, Cu^2+^, Fe^2+^, Hg^2+^, In^3+^, Ni^2+^, Zn^2+^) in the presence of In^3+^ (1 × 10^−4^ M).

Absorption and fluorescence spectral changes and visual color and fluorescence of **SP-IQ** after being placed in the dark for 7 h with various metal cations are shown in Figure 9.

New intense absorption at 410 nm and a 6.4-fold increase in fluorescence at 640 nm are observed only with In^3+^ (Figure 9a,b,e). In the presence of other metal cations except In^3+^, no absorption band at 410 nm and extremely weak fluorescence at 640 nm are observed. Figure 9c shows the yellow solution with the addition of In^3+^ cation, and the colorless or pale pink solutions in the presence of metal cations other than the In^3+^ cation. It was reported that a similar yellow solution of spiropyran derivatives was observed in the presence of H^+^ or CN^−^ [14,18]. Figure 9d shows intense pink fluorescence only with the addition of the In^3+^ cation, while no fluorescence is observed in the presence of other metal cations other than In^3+^. In the dark, upon the addition of the In^3+^ cation, the colorless solution of **SP-IQ** changes slowly to the yellow complex **MCb-IQ–In^3+^** between **MCb-IQ** and In^3+^ cation. Figure 9f shows that the existence of other competing ions does not disturb the selective detection of In^3+^ cation. Selective detection of the In^3+^ cation could be possible with **SP-IQ** by both absorption and fluorescence spectroscopy or with the naked eye.

**Figure 10:**
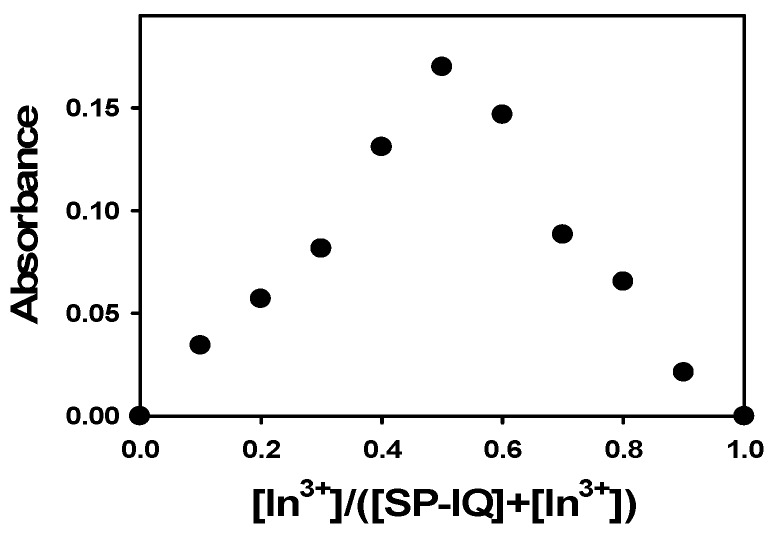
Job plot of **SP-IQ** and In^3+^ cation. Absorbance at 410 nm for the mixed solutions of **SP-IQ** and In^3+^ cation after being placed in the dark for 7 h was a function of the plotted as molar ratio [In^3+^]/([**SP-IQ**]+[In^3+^]). The total concentration for the mixed solutions of **SP-IQ** and In^3+^ cation was 1 × 10^−5^ M.

The Job plot indicates the formation of a 1:1 complex between SP-IQ and In^3+^ cation (Figure 10).

**Figure 11:**
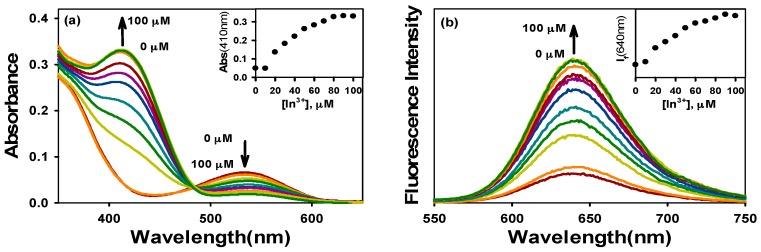
(**a**) Absorption and (**b**) fluorescence spectra of **SP-IQ** after placed in the dark for 7 h with the addition of In^3+^ (0–100 μM, Different color lines represent different In^3+^ concentrations.). Inset: Plots of (**a**) absorbance at 410 nm and (**b**) fluorescence intensity at 640 nm vs. [In^3+^].

Figure 11 shows the absorption and fluorescence spectra of **SP-IQ** after placed in the dark for 7 h with various concentrations of In^3+^ cations. As the concentration of In^3+^ increases, **MC-IQ** formed in the dark converts to **MCb-IQ–In^3+^** complex with In^3+^ cation. Absorption at 540 nm, corresponding to **MC-IQ** decreases, and absorption at 410 nm, corresponding to the **MCb-IQ–In^3+^** complex increases linearly with the increase in concentration of In^3+^. An isosbestic point at 483 nm is clearly observed. Fluorescence intensity at 640 nm increases linearly as the concentration of In^3+^ increases, due to the formation of **MCb-IQ–In^3+^** complex with higher fluorescence efficiency than **MC-IQ**.

The In^3+^ cation could be detected selectively with UV irradiation of **SP-IQ** using absorption and fluorescence spectroscopy in the range of 10–80 μM. The linear detection range of the In^3+^ cation is 10–80 μM and the detection limit of the In^3+^ cation is 10 μM.

Chemosensors for the independent detection of Co^2+^ and In^3+^ cations have been previously reported. However, chemosensors for simultaneous detection of Co^2+^ and In^3+^ cations have not been reported until now (see Table 1). To the best of our knowledge, this is the first report of a dual chemosensor for Co^2+^ and In^3+^ cations.

### 2.5. pH Effect

**Figure 12:**
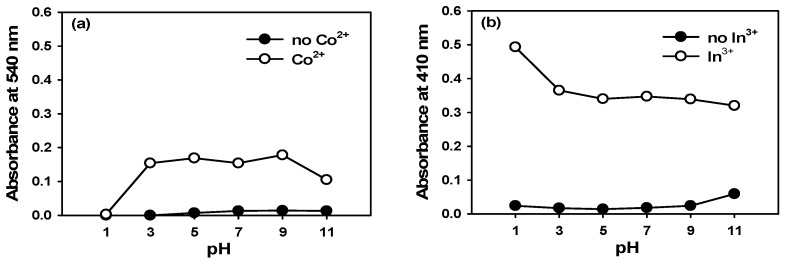
(**a**) Absorbance at 540 nm for **MCa-IQ–Co^2+^** complex and (**b**) absorbance at 410 nm for **MCb-IQ–In^3+^** complex at different pH (1–11).

The effect of pH on the absorption response of the **MCa-IQ–Co^2+^** complex and the **MCb-IQ–In^3+^** complex was investigated in the pH range of 1–11 (Figure 12). The characteristic absorptions of the **MCa-IQ–Co^2+^** complex and the **MCb-IQ–In^3+^** complex are stable between pH 3 and pH 9. Therefore, Co^2+^ and In^3+^ cations could be detected with the naked eye or UV-Vis absorption measurements using the **SP-IQ** chemosensor over a wide pH range of 3–9.

## 3. Materials and Methods

### 3.1. General

The reagents were purchased from Sigma-Aldrich (St. Louis, MO, USA). ^1^H- and ^13^C-NMR spectra were recorded in CDCl_3_ at 400 and 101 MHz, respectively, using an Inova 500 spectrometer (Varian, Palo Alto, CA, USA). UV-Vis absorption spectra were measured using a quartz cuvette in a UV-2401PC spectrophotometer (Shimadzu, Kyoto, Japan). Fluorescence spectra were measured on an AMINCO-Bowman Series 2 spectrofluorometer (Silver Spring, MD, USA). Unless otherwise noted, the concentration of **SP-IQ** is 1 × 10^−5^ M in CH_3_CN/H_2_O (1/1, *v*/*v*). For spectrophotometric titrations, the concentration of Co^2+^ and In^3+^ are used in the range of 0~45 μM and 0–100 μM, respectively.

### 3.2. Synthesis of **SP-IQ**

**Scheme 3:**
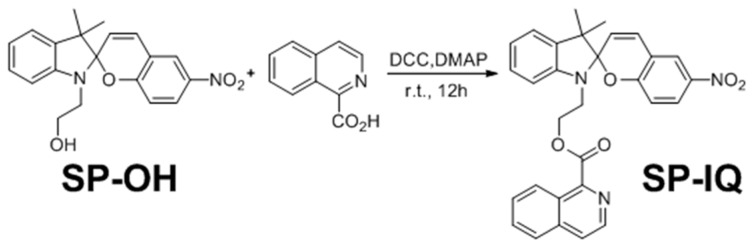
Synthesis of **SP-IQ**.

**SP-IQ**, a spiropyran derivative appended with isoquinoline, was prepared by the reaction between **SP-OH** and isoquinoline-1-carboxylic acid (Scheme 3). **SP-OH** was prepared following the reported procedure [14].

To the mixture of **SP-OH** (100 mg, 0.28 mmol) and isoquinoline-1-carboxylic acid (54 mg, 0.31 mmol) in dichloromethane, dicyclohexylcarbodiimide (58 mg, 0.28 mmol), and 4-dimethylaminopyridine (34 mg, 0.28 mmol) were added at 0 °C. The solution temperature was raised to room temperature and the mixture was stirred for 12 h at room temperature. The reaction mixture was washed with aqueous sodium carbonate solution. The organic layer was dried over anhydrous magnesium sulfate and evaporated. The residual mixture was purified with silica gel chromatography (eluent: ethyl acetate/hexane = 1/1, *v*/*v*). Pure **SP-IQ** (100 mg, 70% yield) was obtained as a light yellow solid.

*2-(3′,3′-dimethyl-6-nitrospiro[chromene-2,2′-indolin]-1′-yl)ethyl isoquinoline-1-carboxylate* (**SP-IQ**). ^1^H-NMR (400 MHz, CDCl_3_). δ 1.26 (s, 3H, -C*H*_3_), 1.27 (s, 3H, -C*H*_3_), 3.68 (m, 1H, -NC*H*_2_CH_2_O-), 3.78 (m, 1H, -NC*H*_2_CH_2_O-), 4.66 (t, 2H, *J* = 4.0 Hz, -NCH_2_C*H_2_*O-), 5.99 (d, 1H, *J* = 10.4 Hz, pyran -C*H*=CHPh-), 6.68 (d, 1H, *J* = 8.8 Hz, NO_2_-Ph *meta*-*H*), 6.78 (d, 1H, *J* = 7.6 Hz, phenyl *H* of indoline), 6.82 (d, 1H, *J* = 10.4 Hz, pyran -CH=C*H*Ph-), 6.89 (t, 1H, *J* = 7.2 Hz, phenyl *H* of indoline), 7.09 (dd, 1H, *J* = 1.2 & 7.6 Hz, phenyl *H* of indoline), 7.19 (dt 1H, *J* = 1.2 & 7.6 Hz, phenyl *H* of indoline), 7.64 (dt, 1H, *J* = 1.2 & 8.4 Hz, phenyl *H* of isoquinoline), 7.74 (dt, 1H, *J* = 1.2 & 8.4 Hz, phenyl *H* of isoquinoline), 7.82 (d, 1H, *J* = 5.4 Hz, pyridyl *meta*-*H* of isoquinoline), 7.84 (s, 1H, NO_2_-Ph *ortho*-*H*), 7.85 (d, 1H, *J* = 8.8 Hz, NO_2_-Ph *ortho*-*H*), 7.88 (d, 1H, *J* = 8.4 Hz, phenyl *H* of isoquinoline), 8.59 (d, 1H, *J* = 5.4 Hz, pyridyl *ortho*-*H* of isoquinoline), 8.69 (d, 1H, *J* = 8.4 Hz, phenyl *H* of isoquinoline). ^13^C-NMR (101 MHz, CDCl_3_). δ 20.0, 26.0, 42.5, 53.1, 63.6, 106.7, 106.9, 115.6, 118.6, 120.1, 122.0, 122.1, 122.7, 124.5, 125.9, 126.4, 126.9, 127.3, 128.1, 128.6, 128.9, 130.8, 136.0, 137.0, 141.1, 141.7, 146.6, 148.3, 159.5, 165.8.

## 4. Conclusions

In summary, the results of the photoinduced interaction between **SP-IQ** and various metal cations are as follows:

Upon UV irradiation, colorless and non-fluorescent **SP-IQ** turns to **MC-IQ** with a pink color, with absorption at 540 nm and fluorescence at 640 nm. Even in the dark, **SP-IQ** converts to **MC-IQ**. In other words, **MC-IQ** is formed from **SP-IQ** by standing in the dark or by UV irradiation. **MC-IQ** returns to **SP-IQ** under room light, i.e., visible light. It is thought that the major isomer under visible light is **SP-IQ**, while the major isomer under UV light or in the dark is **MC-IQ**.

Absorption and fluorescence spectra of **SP-IQ** show no changes with the addition of various metal cations. When UV light is irradiated in the presence of various metal cations, colorless **SP-IQ** forms a pink **MCa-IQ–Co^2+^** complex only with the Co^2+^ cation, which has strong absorption at 540 nm and fluorescence at 640 nm. With other metal cations, absorption at 540 nm and fluorescence at 640 nm are attenuated. In the dark with the addition of various metal cations, **SP-IQ** shows strong absorption at 410 nm and strong fluorescence at 640 nm only with In^3+^ cation, due to the formation of the **MCb-IQ–In^3+^** complex with a yellow color and strong pink fluorescence. From the changes in absorption and fluorescence spectra, it could be roughly deduced that a thermal ring-opening reaction in the ground state favors the zwitterionic structure, which forms the complex with the Co^2+^ cation, whereas the photochemical ring-opening reaction in the excited state favors the quinonoid structure, which forms the complex with the In^3+^ cation [12,13].

The results show that **SP-IQ** acts as a dual sensor for both Co^2+^ and In^3+^ cations. **SP-IQ** could selectively detect Co^2+^ with UV irradiation and In^3+^ cations in the dark, by using absorption and fluorescence spectroscopy or by the naked eye.

## Figures and Tables

**Table 1:** Chemosensors for Co^2+^ and In^3+^ cations.

Structure	Co^2+^ Sensor	In^3+^ Sensor	Other Metal Cation Sensor	Reference (Year)
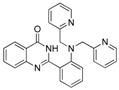	O (Linear range: 3.2 × 10^−8^~1.4 × 10^−6^ M)	X	X	[22] (2008)
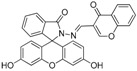	O	X	Ni^2+^	[23] (2011)
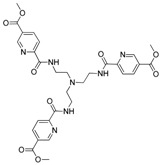	O	X	X	[24] (2014)
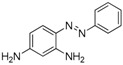	O (Detection Limit: 2 ppm, Linear range: 0.4~1 ppm)	X	X	[25] (2016)
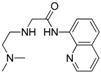	O	X	Zn^2+^	[26] (2016)
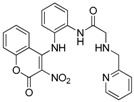	O (Detection Limit: 1.8 × 10^−6^ M)	X	X	[27] (2016)
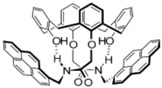	X	O	X	[28] (2005)
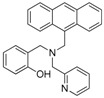	X	O	Al^3+^, Cr^3+^, Fe^3+^, Ga^3+^	[29] (2013)
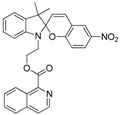	O	O	X	This study
